# Supplementary material for: The Impact of Ethologically Relevant Stressors on Adult Mammalian Neurogenesis
Source: Brain Sci. 2019 Jul 4;9(7):158. doi: 10.3390/brainsci9070158 (PMC6680763; doi:10.3390/brainsci9070158)
Supplement: Supplementary file 1 [file brainsci-09-00158-s001.pdf]

Table. The effects of ethologically relevant stressors on the different stages of adult neurogenesis in several mammalian species

	**Cell Proliferation**		**Cell Survival**		**Cell Differentiation**		
**Stressor**	**Brain region**	**Species**	**Brain region**	**Species**	**Brain region**	**Species**	**Reference**
**Conspecific**							
Separation from peers							
Acute	--		--		--		
Subchronic	↑ DG	♂ California mouse	0 DG	Wistar rat	0 DG	California mouse	[70, 71, 72]
	0 DG	♀ California mouse			0 DG, SVZ	♀ prairie vole	
	0 AMY, Cing, CdPu, DG, HYP	♀ prairie vole					
	↑ SVZ	♀ prairie vole					
Chronic	0 AMY, VMH	♀ prairie vole	0 DG	♂ California mouse	↓ AMY	♀ prairie vole	[70, 71, 72, 73, 74]
	↓ DG, MPOA	♀ prairie vole	↓ DG	♀ California mouse	↔ DG	♀ prairie vole	
			↔ AMY, DG	♀ prairie vole	0 MOB	♀ prairie vole	
			0 Cing, CdPu, HYP, MOB	♀ prairie vole	0 DG	♂ Sprague-Dawley rat	
			↔ DG	♂ Sprague-Dawley rat			
			0 DG	♂ Wistar rat			
			↓ DG	♀ Wistar rat			
Separation from offspring							
Acute	--		--		--		
Intermittent	↓ DG	♀ rat	--		--		[82]
Confrontation via dominance							
Acute	--		--		--		
Subchronic	0 DG	♂ Sprague-Dawley rat	↑ DG	♂ baboon	--		[61, 91]
Chronic	↓ DG	♀ Mole-rat	↑ DG	♂ Sprague-Dawley rat	0 DG	♂ Sprague-Dawley rat	[61, 90, 92]
	↑ DG	♂ Sprague-Dawley rat					
Confrontation via social defeat							
Acute	0 DG	♂ mouse, rat	↓ DG	♂ rat	0 DG	♂ marmoset, tree shrew	[51, 100, 101, 102]
	↓ DG	♂ marmoset, tree shrew			↓ DG	♂ rat	
Subchronic	0 AMY	♂ mouse	↓ DG	♂ mouse, rat	↓ DG	♂ mouse	[103, 104, 105, 106]
	↓ DG	♂ mouse, rat			0 DG	♂ rat	

Chronic	↓ DG	♂ rat, tree shrew	↓ DG	♂ rat	0 DG, PFC	♂ rat	[107, 108, 109, 110, 111]
	↓ PFC	♂ rat	↓ PFC	♂ rat			
	0 SVZ, Mot	♂ rat	0 SVZ, Mot	♂ rat			
Nonconspecific							
Acute (short)	0 DG	♂ rat	↔ DG	♂ rat	0 DG	♂ rat	[115, 116, 117, 118]
Acute (long)	↓ DG	♂ rat					
	0 DG	♀ rat					

acute = one exposure not exceeding 24 hours, subchronic = exposure ranging from 2-10 days, chronic = exposure lasting longer than 10 days

AMY: amygdala; Cing.: cingulate cortex; CdPu: caudate putamen; DG: dentate gyrus of the hippocampus; HYP: hypothalamus; MOB: main olfactory bulb; MPOA: medial preoptic area

Mot: primary motor cortex; SVZ: subventricular zone; VMH: ventromedial hypothalamus

↑: increase; ↓: decrease; 0: no change; ↔: mixed findings; --: no data
